# *Histoplasma* and Cytomegalovirus Coinfection of the Gastrointestinal Tract in a Patient with AIDS: A Case Report and Review of the Literature

**DOI:** 10.3390/diseases5040030

**Published:** 2017-12-08

**Authors:** Jose Armando Gonzales Zamora, Luis Alberto Espinoza

**Affiliations:** 1 Division of Infectious Diseases, Department of Medicine, University of Miami, Miller School of Medicine. Miami, FL 33136, USA; 2 Gilead Sciences, Miami, FL 33136, USA; Luis.Espinoza@gilead.com

**Keywords:** *Histoplasma*, cytomegalovirus, gastrointestinal tract, HIV, AIDS

## Abstract

Opportunistic infections of the gastrointestinal tract are well-documented complications of patients with acquired immunodeficiency syndrome (AIDS). However, concomitant infection by *Histoplasma* and cytomegalovirus has been described rarely. We present the case of an HIV patient with a CD4 count of 20 cells/uL who was admitted with odynophagia and weight loss. Endoscopic evaluation revealed ulcerations in the esophagus and colon, and a mass formation in cecum. Histology revealed budding yeasts in the cecum and a transverse colon consistent with *Histoplasma.* Urine *Histoplasma* antigen was positive. Esophageal tissue disclosed viral cytopathic changes. Immunostaining was positive for cytomegalovirus in the esophagus and transverse colon. The patient was started on appropriate antifungal and antiviral treatment with complete resolution of his symptoms. To our knowledge, this is the fifth case of *Histoplasma* and cytomegalovirus co-infection of the gastrointestinal tract in a patient with AIDS. We also review the literature for similar cases in regards to clinical presentation and the type of gastrointestinal involvement.

## 1. Introduction

The gastrointestinal tract is one of the most common systems affected by opportunistic infections in patients with acquired immunodeficiency syndrome (AIDS). Giardia and cryptosporidium account for the majority of gastrointestinal pathogens isolated in developing countries [1]. However, dimorphic fungal infections gain importance in areas where these organisms are endemic. Histoplasmosis is the most frequent endemic mycosis reported in patients with human immunodeficiency virus (HIV) and may cause disseminated infections in severely immunocompromised patients, affecting the gastrointestinal tract in approximately 12% of cases [2,3]. Another opportunistic pathogen seen in patients with AIDS is cytomegalovirus (CMV), which usually involves the retina, esophagus and colon [4]. The concomitant infection of the gastrointestinal tract by CMV and *Histoplasma* in patients with HIV/AIDS has been rarely described. We present the case of a newly diagnosed HIV-1 patient who developed a gastrointestinal infection caused by these two pathogens. To our knowledge, this is the fifth case reported in the English-language literature. We also review similar cases with regard to clinical presentation and type of gastrointestinal involvement. 

## 2. Case Description

A 31-year-old male, originally from Honduras, was admitted with a two-month history of odynophagia. He also complained of 70-pound weight loss. He denied any past medical history. The patient was a resident of Miami, Florida but reported a recent travel to Los Angeles, California. He admitted having unprotected sexual intercourse with several females. 

**Figure 1:**
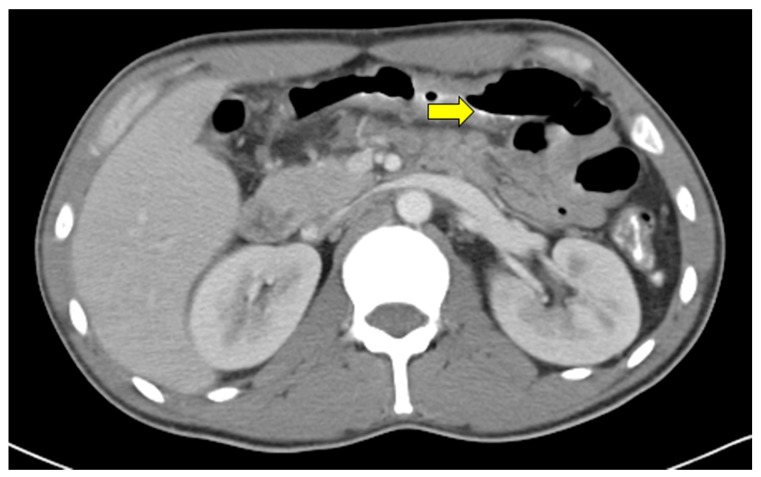
Abdominal computed tomography scan. The yellow arrow points to the wall thickening of the transverse colon.

On admission, his vitals were within normal limits. His physical examination was remarkable due to white plaques in his oral mucosa. The lung, cardiovascular and abdominal exams were normal. He was started on fluconazole for treatment of oropharyngeal candidiasis. On the second day of hospital stay, the patient presented nausea and vomiting. He also developed a fever of 38.4 °C. Laboratory studies revealed a reactive 4th generation ELISA test for HIV. His CD4 count and HIV viral load were 20 cells/uL and 108,000 copies/mL, respectively. Cell blood count revealed leukopenia (2.2 K/uL) and low hemoglobin (10 g/dL). Complementary tests revealed high ferritin (5181 ng/mL) and elevated LDH (1205 unis/L). A chest computed tomography scan was ordered to evaluate for any mechanical esophageal obstruction, but did not reveal any abnormality. Given the presence of the fever and the concern for malignancy, an abdominal CT scan was ordered, which demonstrated focal circumferential bowel wall thickening of the transverse colon (Figure 1). 

**Figure 2:**
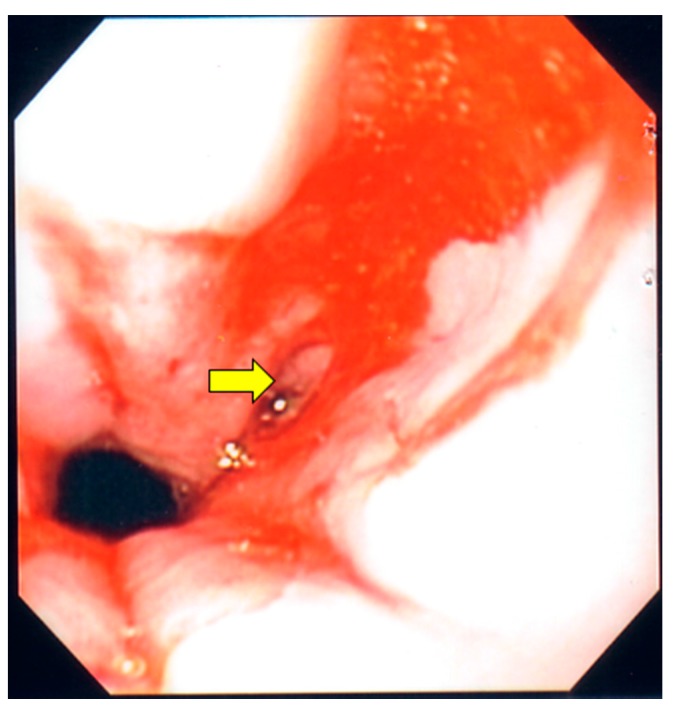
Upper endoscopy. The yellow arrow points to an ulceration found in the mid portion of the esophagus, which was secondary to cytomegalovirus esophagitis.

**Figure 3:**
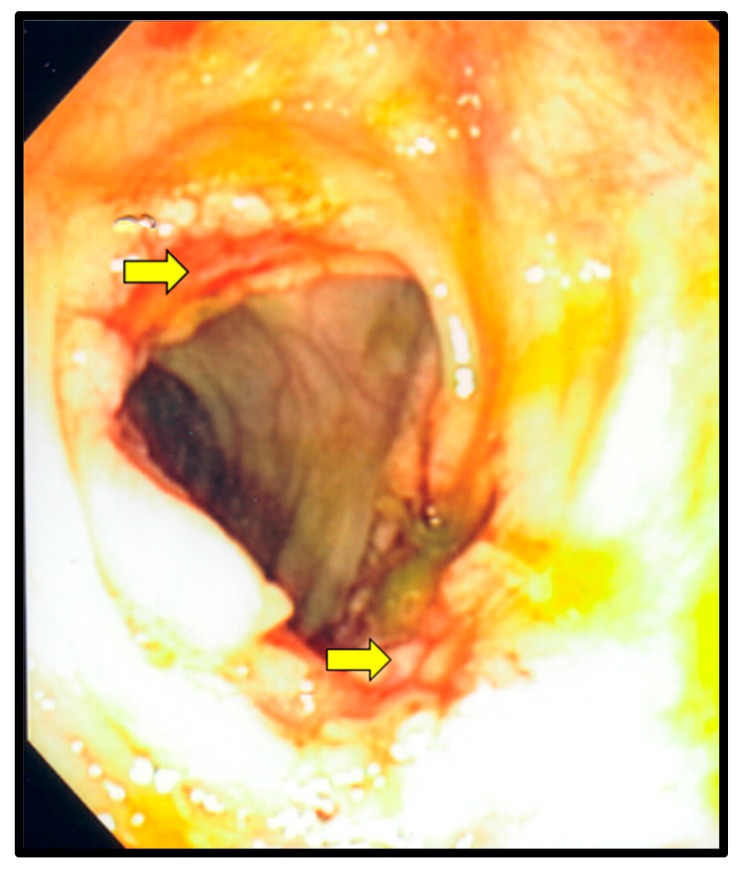
Colonoscopy. The yellow arrows point to ulcerations in the transverse colon, which were secondary to Histoplasmosis and cytomegalovirus co-infection.

**Figure 4:**
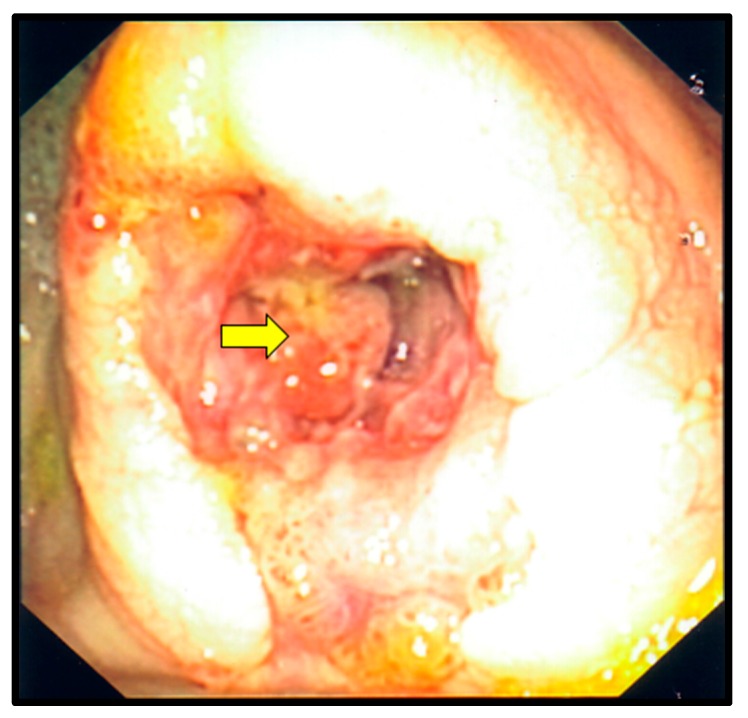
Colonoscopy. The yellow arrow points to a large ulcerative mass in the cecum, which was secondary to gastrointestinal histoplasmosis.

On hospital Day 7, an upper and lower endoscopy were performed. Multiple ulcerations were seen in the mid and distal esophagus (Figure 2). Several ulcerations were also seen in the transverse colon (Figure 3). Additionally, a large ulcerated and friable mass was identified in the cecum (Figure 4). Multiple biopsies were taken. Tissue cultures for fungi, mycobacteria and virus were also ordered. Due to a high suspicion for CMV esophagitis and colitis, the ganciclovir was started. At this point of hospitalization, the urine *Histoplasma* antigen was reported as highly positive (32.33 ng/mL) and Amphotericin was added for possible disseminated histoplasmosis. The following day, an esophageal biopsy disclosed ulcerated squamous mucosa with viral cytopathic changes in the lamina propria, suggestive of CMV infection. Moreover, biopsies from cecal mass and the transverse colon revealed intracellular budding yeasts on Gomori methanamine stain consistent with *Histoplasma capsulatum.* Immunostaining of transverse colon tissue was positive for CMV. 

**Figure 5:**
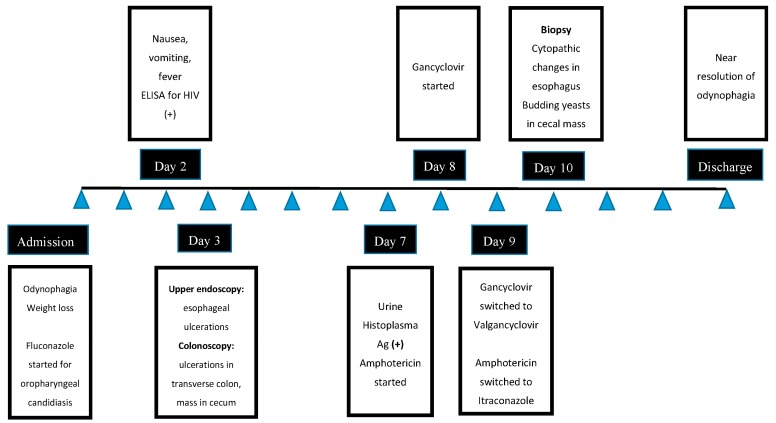
Flowchart of hospitalization course.

After three days of treatment, the patient experienced significant improvement of odynophagia and was able to tolerate oral medications. The fever also resolved. Ganciclovir was switched to oral valgancyclovir 900 mg, twice daily, and amphotericin was changed to oral itraconazole 200 mg, twice a day. Prophylaxis with trimethoprim/sulfamethoxazole and azithromycin was also initiated. He was discharged in a stable condition with a close clinic follow-up (Figure 5). He was seen as an outpatient two weeks later and reported a complete resolution of odynophagia and a 25-pound weight gain. Antiretroviral therapy with tenofovir/emtricitabine/efavirenz was initiated on that visit. At a three-month follow up, his CD4 count increased to 122 cells/uL and his HIV viral load decreased to 128 copies/mL. He completed a six-week course of oral valgancyclovir 900 mg, twice a day, and then it was switched to a maintenance dose of 900 mg, daily, for six months. Treatment with itraconazole was given for one year.

## 3. Discussion

Histoplasmosis is an increasingly recognized opportunistic infection in patients with AIDS who reside in endemic areas. Severely immunocompromised patients are at high risk of developing a disseminated form of this infection, which occur in almost 95% of cases [2]. The widespread dissemination of organisms in these patients can affect any organ system including the gastrointestinal tract, which can be involved in up to 12% of cases [3]. The most frequently reported symptoms in HIV patients with gastrointestinal histoplasmosis include fever, diarrhea, weight loss and abdominal pain [2]. According to some case series, the colon and cecum are the most commonly involved sites, whereas in immunocompetent individuals, disease of the terminal ileum predominates [2,5]. The endoscopic features found among AIDS patient with gastrointestinal histoplasmosis include ulcerations, pseudopolyps, plaques and a thickened wall. Ulcerations are reported in the majority of cases (85.7%). Masses mimicking colon cancer have been rarely described [5]. The mortality of gastrointestinal histoplasmosis in the setting of HIV is high, but improvements on antiretroviral therapy seems to have caused a favorable impact in prognosis [2].

Another infection commonly seen among AIDS patients is CMV. In individuals with a CD4 count below 50 cells/uL, reactivation of CMV can lead to localized infections in one or multiple organs. According to some reports, the retina is the leading affected organ, followed by the gastrointestinal tract (17% of cases). In this latter system, the esophagus and colon are the most frequent sites of involvement [4]. This prognosis of gastrointestinal CMV infection has also improved significantly since the introduction of antiretroviral therapy and nowadays it is an infrequent infection mostly restricted to HIV patients with a late diagnosis or with poor compliance to antiretroviral therapy [6].

CMV gastrointestinal infection can also occur along with other opportunistic pathogens. Candida is the most common organism found in a concomitant manner with CMV, usually causing esophagitis [7]. Other pathogens described in association with CMV have been mycobacterium tuberculosis, mycobacterium avium and adenovirus [8]. Our patient developed CMV and *Histoplasma* co-infection of the gastrointestinal tract, which has been rarely reported in the literature. 

To review our current state of knowledge on this subject, we searched MEDLINE (1946 to May 2017) via OVID and EMBASE (1967 to May 2017) via Scopus for the relevant Medical Subject Headings terms in English-language literature. We included only the cases in which *Histoplasma* capsulatum and cytomegalovirus were identified simultaneously as a cause of gastrointestinal infection in patients with HIV. We also searched within references of these case reports for relevant articles. We found four case reports fitting our search criteria (Table 1) [9,10,11,12]. The mean age of affliction was 44.25 years, ranging from 40 to 51 years. All the cases were in males. The mean CD4 count was 18.6 cells/uL, ranging from 8 cells/uL to 31 cells/uL. The most common symptoms on presentation were fever and weight loss, which were reported on two occasions in each one. The colon was affected by *Histoplasma* and cytomegalovirus in two patients [10,11]. One patient presented involvement of the ileum by these two organisms [12]. The oral mucosa was affected by *Histoplasma* and CMV in one patient, who also presented CMV infection in the stomach [9]. Only two patients had extraintestinal involvement [9,12]. In one of them, CMV caused a disseminated infection involving the lungs, adrenal glands and spleen [9]. The other patient with extraintestinal manifestations presented only lung involvement, of which an etiologic pathogen was not specified in the report [12]. The outcome was favorable in two patients [11,12]. One patient expired secondary to a disseminated CMV infection [9].

We believe our case is unique because gastrointestinal histoplasmosis manifested in the colon with two different type of lesions, ulcerations and a mass formation. This presentation could have been easily confounded with a malignant process. *Histoplasma* was ultimately identified by histologic examination, however, a highly positive urine *Histoplasma* antigen allowed an early diagnosis and treatment, which probably contributed to the rapid recovery of our patient. On the other hand, CMV was suspected early in the course of the hospitalization given the presence of esophageal ulcerations in a patient not responding to fluconazole therapy. CMV infection was later confirmed by histology and immunostaining. Another interesting feature of our patient was the identification of CMV in two distant sites of the gastrointestinal tract: the esophagus and the colon.

## 4. Conclusions

Gastrointestinal *Histoplasma* and CMV coinfection is very rare, even in the setting of AIDS. This case highlights the importance of considering histoplasmosis and CMV as part of the differential diagnosis of colonic masses and ulcerations. Clinicians should be aware that concomitant infections can be found in severely immunocompromised patients, thus, a complete evaluation is always warranted.

## Figures and Tables

**Table 1:** Summary of Cases Reported in the Literature for CMV and *Histoplasma* Co-infection of the gastrointestinal tract in patients with HIV/AIDS.

Patient	Report	Age	Sex	Symptoms	CD4 Count (Cells/uL)	Affected GI * Area by *Histoplasma*	Affected GI Area by CMV	Type of GI Lesions	Extraintestinal Organs Involved	Outcome
1	Jones et al (1992) [2]	51	M	Painful oral ulcerations	Unknown	Oral mucosa	Oral mucosa, stomach	Ulcerations	Lungs, adrenal glands, spleen ^a^	Died
2	Fan X et al. (2008) [3]	45	M	Abdominal pain, weight loss, fever	17	Ascending colon	Ascending colon	Mass	None	Unknown
3	Khara H et al. (2013) [4]	41	M	Diarrhea, weight loss, fever	31	Descending colon	Descending colon	Ulcerations	None	Survived
4	Bruno MA et al. (2016) [5]	40	M	Odynophagia, cough, night sweats, dyspnea, GI bleeding	8	Ileum	Ileum	Mass	Lungs ^b^	Survived
5	Present report	31	M	Odynophagia, weight loss	20	Cecum, transverse colon	Esophagus, transverse colon	Mass, ulcerations	None	Survived

* GI = gastrointestinal. ^a^ Lungs, adrenal glands and spleen were involved by CMV. ^b^ Organism that caused lung involvement was not specified in the report.
